# Exome Sequencing Identifies *ZNF644* Mutations in High Myopia

**DOI:** 10.1371/journal.pgen.1002084

**Published:** 2011-06-09

**Authors:** Yi Shi, Yingrui Li, Dingding Zhang, Hao Zhang, Yuanfeng Li, Fang Lu, Xiaoqi Liu, Fei He, Bo Gong, Li Cai, Ruiqiang Li, Shihuang Liao, Shi Ma, He Lin, Jing Cheng, Hancheng Zheng, Ying Shan, Bin Chen, Jianbin Hu, Xin Jin, Peiquan Zhao, Yiye Chen, Yong Zhang, Ying Lin, Xi Li, Yingchuan Fan, Huanming Yang, Jun Wang, Zhenglin Yang

**Affiliations:** 1 The Sichuan Provincial Key Laboratory for Human Disease Gene Study, Sichuan Academy of Medical Sciences and Sichuan Provincial People's Hospital, Chengdu, Sichuan, China; 2 Institute of Laboratory Medicine, Sichuan Academy of Medical Sciences and Sichuan Provincial People's Hospital, Chengdu, Sichuan, China; 3 Beijing Genome Institute at Shenzhen, Shenzhen, China; 4 Department of Ophthalmology, Sichuan Academy of Medical Sciences and Sichuan Provincial People's Hospital, Chengdu, Sichuan, China; 5 Innovative Program for Undergraduate Students, School of Bioscience and Biotechnology, South China University of Technology, Guangzhou, China; 6 The Department of Ophthalmology, Xinhua Hospital, Shanghai Jiaotong University, Shanghai, China; Yale University, United States of America

## Abstract

Myopia is the most common ocular disorder worldwide, and high myopia in particular is one of the leading causes of blindness. Genetic factors play a critical role in the development of myopia, especially high myopia. Recently, the exome sequencing approach has been successfully used for the disease gene identification of Mendelian disorders. Here we show a successful application of exome sequencing to identify a gene for an autosomal dominant disorder, and we have identified a gene potentially responsible for high myopia in a monogenic form. We captured exomes of two affected individuals from a Han Chinese family with high myopia and performed sequencing analysis by a second-generation sequencer with a mean coverage of 30× and sufficient depth to call variants at ∼97% of each targeted exome. The shared genetic variants of these two affected individuals in the family being studied were filtered against the 1000 Genomes Project and the dbSNP131 database. A mutation A672G in *zinc finger protein 644 isoform 1* (*ZNF644*) was identified as being related to the phenotype of this family. After we performed sequencing analysis of the exons in the *ZNF644* gene in 300 sporadic cases of high myopia, we identified an additional five mutations (I587V, R680G, C699Y, 3′UTR+12 C>G, and 3′UTR+592 G>A) in 11 different patients. All these mutations were absent in 600 normal controls. The *ZNF644* gene was expressed in human retinal and retinal pigment epithelium (RPE). Given that *ZNF644* is predicted to be a transcription factor that may regulate genes involved in eye development, mutation may cause the axial elongation of eyeball found in high myopia patients. Our results suggest that *ZNF644* might be a causal gene for high myopia in a monogenic form.

## Author Summary

People with myopia see near objects more clearly than objects far away. Myopia is the most common ocular disorder worldwide, with a high prevalence in Asian (40%–70%) and Caucasian (20%–30%) populations. Although the etiologies of myopia have not yet been established, previous studies have indicated the involvement of genetic and environmental factors (such as close working habits, higher education levels, and higher socioeconomic class). Genetic factors play a critical role in the development of myopia, especially high myopia. In this study, we use exome sequencing, a powerful tool for a disease gene identification, to identify a gene involved in high myopia in a monogenic form among Han Chinese. Mutations in *zinc finger protein 644 isoform 1* (*ZNF644*) were identified as potentially responsible for the phenotype of high myopia. The main feature of high myopia is axial elongation of the eye globe. Given that *ZNF644* is predicted to be a transcription factor that may regulate genes involved in eye development, a mutant ZNF644 protein may impact the normal eye development and therefore may underlie the axial elongation of the eye globe in high myopia patients. Further study of the biological function of *ZNF644* will provide insight into the pathogenesis of myopia.

## Introduction

Myopia is the most common ocular disorder worldwide, with a prevalence of 20–30% in North American, European and Australian populations [1], [2] and as high as 40–70% in the Asian population [3]–[5]. One type of myopia is high myopia, and it is prevalent in 1–2% in the general population [1]–[6].

In high myopia, affected patients' eyes have a spherical equivalent of less than or equal to −6.00 diopter sphere (DS) and an axial length longer than or equal to 26.0 mm. In some cases, high myopia may also show retinal pathological changes with progressive choroidal degeneration in the posterior pole and other complications, potentially resulting in severe vision loss. In such cases, high myopia is referred to as pathological or degenerative myopia, which is one of the leading causes of blindness in the world [1], [7], [8].

The exact pathogenesis of myopia remains unclear. There are indications that environmental factors (such as close working habits, higher education levels and higher socioeconomic class) [9], [10] and genetic predisposition both contribute to the development of myopia [10], [11], especially of high myopia [12]. The evidence that genetic variation plays a crucial role in the occurrence and development of myopia is based on studies showing different frequencies of myopia in different populations [2]–[5], [13], obvious family aggregation trends, twin studies [9], [14], [15], and the identification of 18 linked loci having an association with myopia (OMIM, 160700) [6], [16]–[19].

Myopia can be inherited as a complex trait or in a monogenic form. For the complex form, myopia appears to be the result of an interaction of multiple genes and environmental factors. Recently, several loci have been identified by genome-wide association study (GWAS) as being responsible for complex myopia [17]–[20]. On the other hand, high myopia in a monogenic form may be inherited in an autosomal dominant, autosomal recessive and X-linked recessive manner [15], [21]. In this study, we propose to use exome sequencing to identify a gene responsible for high myopia in a monogenic form in a Han Chinese population.

## Results

**Figure 1:**
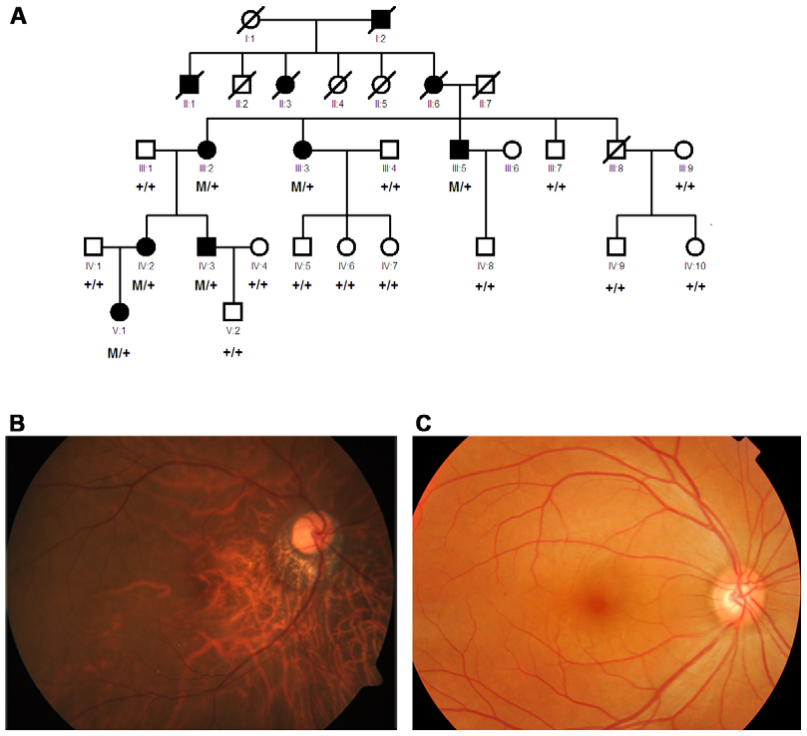
Pedigree and segregation of the mutation and fundus photograph of a patient from the family. A. Kindred structure and segregation of *ZNF664* S672G mutation in the high myopia family. Affected individuals are shown by solid squares (males) or circles (females). Normal individuals are identified by open symbols. Deceased individuals are indicated by a slash (/). M: 672G mutant allele of *ZNF644*; +: S672 normal allele of *ZNF644*. B. Fundus photograph of III:2 of the family, showing tigroid or tessellated features and conus pattern of retina. C. Normal fundus appearance of IV:5.

Here we describe a Han Chinese family (951) from Chengdu, China, that has monogenic high myopia with a dominant inheritance model. The clinical features of the nineteen of 20 living family members who participated in this study are shown in Table 1. Ten patients within the family were diagnosed with high myopia; six were alive and available for this study. The four deceased patients were diagnosed based on available medical records (Figure 1A). The six living had refractive errors ranging from −6.27 to −20.00 diopter sphere (DS) for the left eye (OS) and from 7.51 to 11.49 DS for the right eye (OD), and eye globe axial length ranging from 26.0 to 31.1 mm for OS and 26.9 to 30.5 mm for OD. They also had pre-school age of onset. Three elderly patients showed typical fundus features of high myopia: a thinning of the RPE and the choriocapillaris, which gives what is described as a ‘tigroid’ or ‘tessellated’ fundus appearance (Figure 1B, Table 1).

**Table 1:** The clinical features of the high myopia family 951 with a *ZNF644* gene mutation.

Subject No.	Age (Yr)*	Gender	Axial length (mm)**		Refractive errors (DS)**		Fundus appearance**		ZNF644 mutation***	
			OD	OS	OD	OS	OD	OS	Nucleotide change	Amino acid change
III:1	71	M	23.8	23.9	0	−0.22	normal	normal	2156 A	S672
III:2	68	F	28.6	29.5	−11.5	−13.02	tigroid or tesselated	tigroid or tesselated	2156 A>G	S672G
III:3	73	F	30.5	31.1	−16.3	−20.04	tigroid or tesselated	tigroid or tesselated	2156 A>G	S672G
III:4	74	M	24	24.3	0	0	normal	normal	2156 A	S672
III:5	65	M	27.1	28.2	−9.27	−11.02	tigroid or tesselated	tigroid or tesselated	2156 A>G	S672G
III:7	70	M	23.7	24	−0.5	−0.5	normal	normal	2156 A	S672
III:9	65	F	23.9	24.1	0.23	0	normal	normal	2156 A	S672
IV:1	38	M	24.5	24.3	0	0	normal	normal	2156 A	S672
IV:2	27	F	26.5	27.2	−7.53	−9.49	normal	normal	2156 A>G	S672G
IV:3	25	M	28.9	29.4	−12.11	−12.31	normal	normal	2156 A>G	S672G
IV:4	32	F	24.1	25.2	−0.38	−0.5	normal	normal	2156 A	S672
IV:5	36	M	24.9	24.8	−0.46	−0.42	normal	normal	2156 A	S672
IV:6	34	F	24.8	25	−0.48	−0.5	normal	normal	2156 A	S672
IV:7	31	F	24.8	24.9	−0.32	−0.44	normal	normal	2156 A	S672
IV:8	34	M	24.3	24.2	−0.2	0.2	normal	normal	2156 A	S672
IV:9	36	M	23.9	23.8	0.25	0.4	normal	normal	2156 A	S672
IV:10	30	F	24.9	25.2	−0.1	−0.12	normal	normal	2156 A	S672
V:1	8	F	26.9	26	−9.54	−6.34	normal	normal	2156 A>G	S672G
V:2	6	M	24.3	24.2	0.22	0.15	normal	normal	2156 A	S672

*The age when the family member was recruited in 2009.

**OS, left eye; OD, right eye; DS, diopter sphere.

***Location: chromosome 1: 9117748 (exon 3 of *ZNF644* gene).

For this analysis, we selected two affected family members (V:1 and III:2) (Figure 1A, Table 1). V:1, the proband, at age 8 in 2009, had refractive errors at −9.54 DS (OD) and −6.34 DS (OS) and normal fundus features. III:2, at age 68 in 2009, had refractive errors at −11.49 DS (OD) and −13.02 DS (OS) and tessellated or tigroid features (Table 1, Figure 1B).

**Figure 2:**
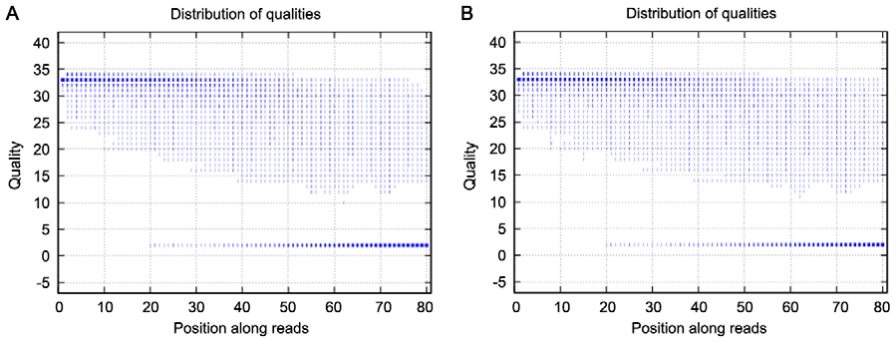
The distribution of qualities of the sequencing reads for the two analyzed samples. A. The sequencing quality of III:2. B. The sequencing quality of V:1. The X-axis represents the position along each sequence read. In this study we performed 80 bp sequencing, so the label of X-axis is from 0 to 80. The Y-axis is the Phred quality score of each base along sequence reads. The higher the score, the more accurate a base call. The quality scores of 20, 30 and 40 represent 99%, 99.9% and 99.99% accuracy of a base call. The intensity of the blue dot refers to the quantity of sequences with the same quality score. The darker the blue, the more bases/sequences at the dot. The quality scores of first 50 bp of most reads for both samples are higher than those of the last 30 bp, thus the darker blue dots accumulate around Q30 (Phred score of 30) from 0 to 30 bp along reads, while Q5 is observed between 60∼80 bp along reads. In order to get more accurate base calling, we filtered these low quality reads during the data analysis.

We used exome sequencing to identify potential variants responsible for high myopia in this family. We generated an average of 2.4 Gb of sequence with 30× average coverage for each individual as single-end, 80-bp reads, and about 97% (∼32.98 Mb in length) of the targeted bases were covered sufficiently to pass our thresholds for calling SNPs and short insertions or deletions (indels) (Table 2). The bases with quality scores above 20 (99% accuracy of a base call) represent over 75% of total sequence data, while the error rate is below 3% (Figure 2). Table 3 presents the exome genetic variants identified from the exome sequencing analysis. The numbers in Table 3 are comparable to what was reported in two previously published results [4], [20]. The transition versus transversion ratio is 2.95 and 2.69 for the two samples respectively. The rate of heterozygous versus homozygous variants is 1.33 and 1.38 for the two samples respectively. For patients V:1 and III:2, respectively, we identified 10,156 and 10,358 SNPs (synonymous and non-synonymous) in coding regions; 447 and 501 variants (SNPs and indels) in introns that may affect splicing (within 5 bp of the intron/exon junction); and 2,370 and 2,642 indels in coding regions or introns. Given that these patients are related and they are expected to share the causal variant for high myopia, we filtered all the detected variations in these patients against each other and found that they shared 6,610 variants (SNPs and indels) (Table 3).

**Table 2:** Summary statistics for exome sequencing for two individuals with high myopia.

Sequencing reads				Called coverage		
Total	Unique mapping	Overlapping target/unique	Nonduplicated	Mean coverage	Called bases	% of CCDS*
23,944,773	22,480,147	8,324,968	7,669,803	96.17%	636,987,416	87.28
28,792,273	27,118,103	10,088,253	9,215,629	97.16%	776,725,130	88.69

*CCDS: consensus coding sequence;

%CCDS represents the number of CCDS with over 80% coverage of the total number of CCDS.

**Table 3:** Genetic variants identified through exome resequencing.

Filter	Genetic variants				
	Synonymous SNP	Non-synonymous SNP	Splice acceptor and donor site	Indel	Total
V:1	5723	4433	447	2370	12973
III:2	5810	4548	501	2642	12701
V:1 and III:2 Shared	2823	2138	243	1406	6610
Not in 30 CHB* data of 1000 Genomes Project or dbSNP131	61	62	5	265	393

*CHB, Han Chinese Beijing.

Because high myopia is a rare disorder but has a clear phenotype, there is a very low likelihood of the causal mutation in these patients being shared with a wider healthy population. We therefore compared the shared variants in these patients with the Han Chinese Beijing SNPs from dbSNP131 and the data from 30 genomes of Han Chinese Beijing recently available from the 1000 Genome Project (February 28, 2011 releases for SNPs and February 16, 2011 releases for indel fttp://www.1000genome.org). This left a total of 393 variants that were shared between these two patients. Of these, 332 genetic variants (including 62 non-synonymous SNPs, 5 splice acceptor and donor sites, and 265 indels) were predicted to potentially have a functional impact on the gene (Table 3). We carried out Sanger sequencing validation on these 332 variants, and obtained accuracy of 98% (66/67) for called SNPs and 96% (254/265) for indels, indicating the high quality of our variant calling method.

**Figure 3:**
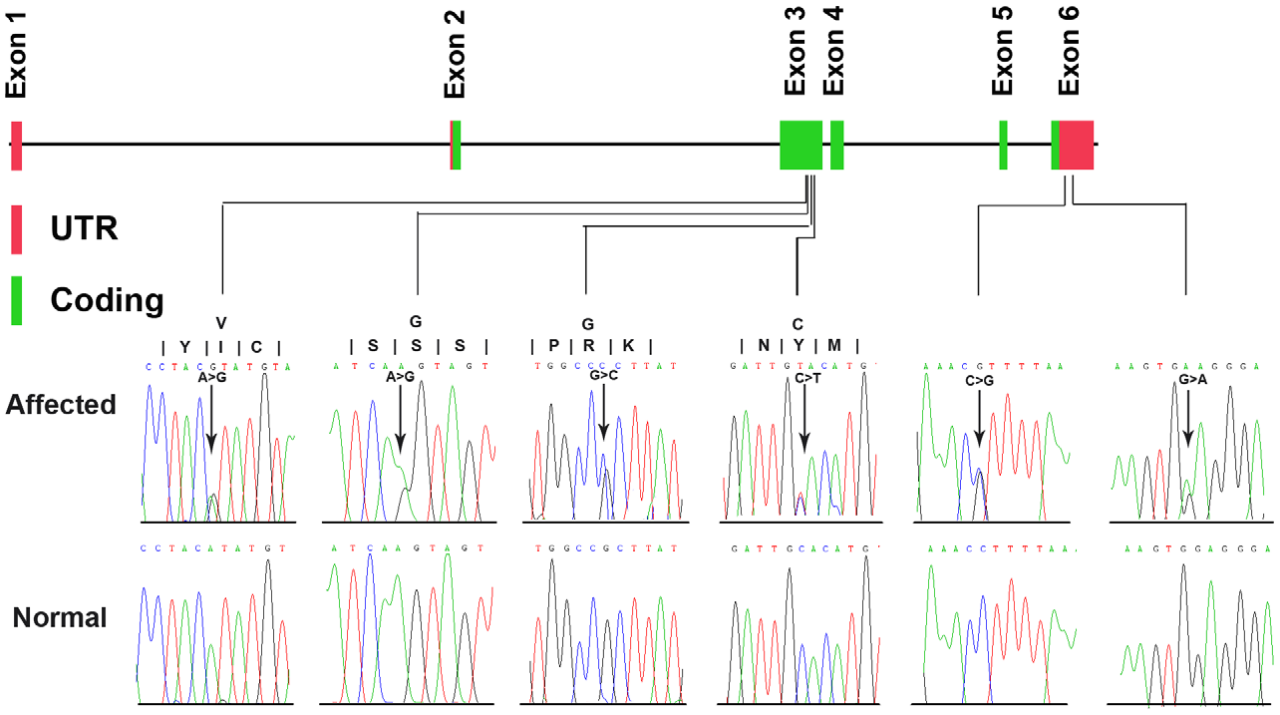
Genomic structure of the exons encoding the open reading frame of *ZNF644* and identified mutations. Five out of six exons are translated (green), and exon 1 and portions of exon 2 and exon 6 are untranslated (red) in the *ZNF644* gene (upper panel). Six different mutations in the *ZNF644* gene and their sequencing traces are shown at the bottom of the figure (lower panel).

We then performed segregation analysis by Sanger sequencing on the 66 validated SNPs and 254 indels, using the available 19 members of family 951 (Figure 1). Only one variant co-segregated with the disease phenotype in this family: an A to G change in exon 3 (2156A>G), resulting in an S672G amino acid change, in the *zinc finger protein 644 gene isoform 1* (*ZNF644*, located at 1p22.2) (Table 1, Figure 1, Figure 3). We obtained a LOD score of 3.19 at theta = 0 given an autosomal dominant mode of inheritance with full penetrance and 0.0001 for the disease allele frequency. The power to obtain a LOD score greater than 3 was 88% when tested by SLINK, providing further support for this mutation being the disease-causing change for family 951. We then assessed the presence of the co-segregating mutation in the 600 matched normal controls using direct PCR sequencing of the *ZNF644* exon 3, and did not find it in the 600 controls.

**Figure 4:**
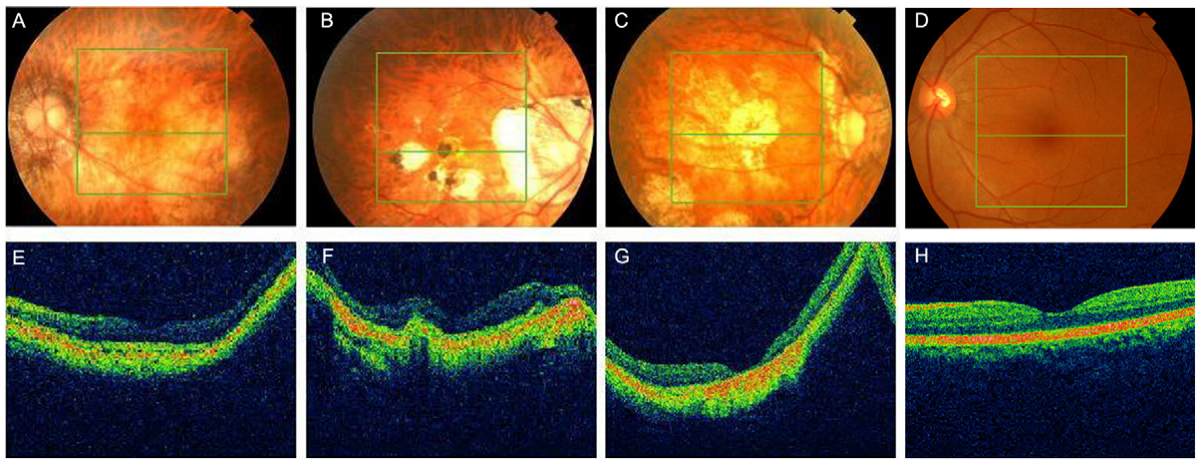
Fundus photographs and optical coherence tomography (OCT) of high myopia patients from the sporadic cases. A. The fundus of the patient JS047001 (Table 5) showing tigroid or tessellated features, conus, and CNV (choroid neovascularization). Optical Coherence Tomography (OCT) examination of this patient showed continuity of retinal pigment epithelial layer and broken photoreceptor layer (E). B. The fundus of the patient JS103001 (Table 5) showing tigroid or tessellated features, numerous areas of atrophy of the pigment epithelium, and choriocapillaries extending into the macular region and Fuchs spot. OCT examination of this patient showed discontinuity and irregular apophysis of the reflective pigment epithelial layer (F). C. The fundus of the patient JS104001 (Table 5) showing tigroid or tessellated features, numerous areas of atrophy of the pigment epithelium, and choriocapillaris. OCT examination of this patient showed foveal thinning and atrophies of the retinal neuroepithelial layer (G). D. Normal fundus photograph and OCT examination of a normal Control (H).

We further carried out direct PCR sequencing of the *ZNF644* exons in an additional 300 unrelated (based on their self-identified geographical ancestry), sporadic high myopia patients. The 300 patients had refractive errors ranging from −6.0 to −29.0 DS for both eyes and an axial eye globe length from 26 to 33 mm for both eyes (Table 4). Some of these individuals also showed severe retinal pathological changes in the fundus appearance, an abnormal RPE, and photoreceptor layer alterations at the time of the OCT examination (Figure 4, Table 5). In the 300 sporadic patients, we identified a total of 8 variants when we sequenced the *ZNF644* exons, and five out of these 8 variants (present in 11 unrelated individuals) were absent in all the 600 controls. Among these five mutations identified from the sporadic cases, three were in exon 3 (I587V, R680G and C699Y) and two were in the 3′ untranslated region (UTR) (+12 C>G and +592 G>A) (Figure 3, Table 5). The remaining three out of the 8 variants were found in both cases and controls, two (T404T and V444V) were synonymous changes which may not affect the biological function of *ZNF644* and one was located in the 3′UTR (+1015 C>G). The *P*-value for the 17 potentially functional variants in 301 patients with high myopia (One mutation identified in one member of the high myopia family 951 plus the 6 mutations identified in the 16 unrelated patients from the 300 sporadic cases) compared with these variants being seen in 600 controls (3 in 600 controls) was 2.28×10^−6^ by Fisher's exact test. This data suggests that there are multiple rare variants in *ZNF644* associated with high myopia.

**Table 4:** Characteristics of sporadic cases and controls in the study.

	Number	Age (Yr)*	Gender		Refractive errors (Diopter)**	Axial length (mm)**
			Male	Female		
**Cases**	300	33.65±12.66	139	161	−10.54±4.23 (OD), −10.36±4.03 (OS)	28.02±2.02 (OD), 28.00±2.03 (OS)
**Controls**	600	55.85±9.06	287	313	−0.02±0.02 (OD), −0.01±0.01 (OS)	23.91±1.98 (OD), 24.02±2.02 (OS)

*The age when the cases and controls were recruited;

**OD: right eye; OS: left eye.

**Table 5:** The clinical features of affected patients with a *ZNF644* gene mutation in the 300 sporadic cases with high myopia.

Family	Subject No.	Age (Yr)*	Gender	Axial length (mm)**		Refractive errors (DS)**		Fundus appearance**		*ZNF644* mutation			
				OD	OS	OD	OS	OD	OS	Nucleotide change	Exon	Amino acid change	Chr.1 location
JS007	JS007001	26	M	26.1	26.1	−10.02	−9.47	conus	conus	2091 A>G	3	I587V	91177740
JS103	JS103001	55	F	29.1	29.2	−14.47	−15.03	tigroid, CA, Fuchs spot	tigroid, CA, Fuchs spot	2091 A>G	3	I587V	91177740
JS104	JS104001	53	M	31.5	29.4	−17.25	−16.49	tigroid, CA	tigroid, CA, Fuchs spot	2091 A>G	3	I587V	91177740
JS078	JS078001	28	F	27.8	27.0	−12.03	−9.47	tigroid, macular atrophy	tigroid or tesselated	2091 A>G	3	I587V	91177740
JS131	JS131001	31	M	27.2	27.1	−14.67	−15.04	tigroid or tesselated	tigroid	2091 A>G	3	I587V	91177740
JS010	JS010001	29	M	26.5	26.1	−10.52	−9.54	conus	conus	2180 C>G	3	R680G	91177461
JS064	JS064001	32	F	28.4	28.4	−9.54	−10.97	tigroid, conus	tigroid	2238 G>A	3	C699Y	91177403
JS080	JS080001	25	F	26.8	27.0	−11.26	−11.45	tigroid	tigroid	2238 G>A	3	C699Y	91177403
JS075	JS075001	19	M	30.5	28.5	−18.02	−15.02	tigroid, conus	tigroid	4138 C>G	6	3′UTR+12 C>G	91154931
JS027	JS027001	63	F	26.3	26.5	−8.02	−9.04	tigroid, conus	tigroid, conus	4718 G>A	6	3′UTR+592 G>A	91154351
JS047	JS047001	46	M	28.5	28.7	−15.96	−16.02	tesselated, conus, CNV	tesselated, conus	4718 G>A	6	3′UTR+592 G>A	91154351

*The age when the patients were recruited.

**OD: right eye; OS: left eye; DS, diopter sphere; CA: choriocapillaris atrophy; CNV: choroidal neovascularization.

**Figure 5:**
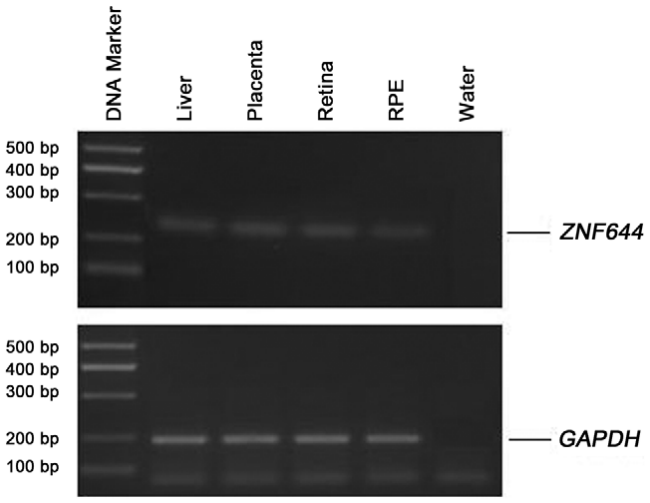
Expression of the *ZNF644* gene in human tissues. RT-PCR analyses of *ZNF644* expression in the human liver, placenta, retina, and retinal pigment epithelium (RPE) with 255 bp of products. *GAPDH* was used as an internal control for cDNA quantification.

To make sure that the *ZNF644* gene is expressed in the eye, we examined *ZNF644* expression in different human tissues using reverse transcript polymerase chain reaction (RT-PCR). The *ZNF644* gene was expressed in the human retina and RPE as well as in the liver and placenta (Figure 5).

## Discussion

For the past several decades, standard methods for identifying genes underlying disease in a monogenic form have primarily been through selecting candidate genes for testing or by using positional cloning. The candidate gene approach requires less work and costs less because only the candidate gene needs to be sequenced, but this method requires prior knowledge of the pathogenesis of a disease for gene selection. This fundamentally impedes the disease gene identification speed because the pathogeneses of many diseases have not yet been unmasked. Without pathogenesis information of a disease, the traditional positional cloning strategy can be used first to map the disease gene in the chromosome and then to identify the disease-causing gene within a specific interval. Thus the pathogenesis of a disease can be explored based on the identified disease gene. However, the positional cloning method requires marked locus heterogeneity and the availability of a large family. Focusing on the exome can be especially fruitful in disease gene identification given that previous studies have indicated that approximately 85% of causal mutations for human diseases are located within the coding region and canonical splice acceptor and donor sites (http://www.hgmd.cf.ac.uk/ac/index.php). Therefore, through sequencing and comparing the coding region of affected and unaffected individuals within a family and filtering the benign changes using a public database, such as 1000 Genomes Project and dbSNP databases, the mutation in the coding region can be identified even within small families and without knowing the pathway of a disease and marked locus heterogeneity. Currently, the cost of the exome sequencing method is even less than that of the positional cloning strategy. So, this method will not only speed up disease gene identification but will enable us to systematically tackle previously intractable monogenic disorders. In fact, exome sequencing approaches have been successfully used to identify disease genes for Mendelian disorders in recent studies [21]–[36]. Unfortunately, compared to the positional cloning strategy, the exome sequencing method may not identify the mutations in non-coding regions. This limitation promotes the use of the whole sequencing method to identify disease genes [26], [37]. Theoretically the whole gene sequencing will eventually become the best method of disease gene identification, because this method has the advantages of both positional cloning and exome sequencing methods. It can also identify disease genes caused by a large indel, inversion, translocation, and other chromosome structure aberrant. However, at the current stage, whole genome sequencing costs more and needs a lot of bioinformatics work, and this restricts its use in disease gene identification. Presently exome sequencing is a powerful tool with low cost for identifying genes that underlie disease. The whole genome sequencing method will very likely become the most powerful method for disease gene identification as the constant improvements to massively sequencing technologies and the impending massively parallel single-molecule sequencing technologies will reduce method costs and time barriers [38]. Practically, candidate gene approach, positional cloning strategy and exome sequencing or whole genome sequencing methods has been combined to identify the disease-causing genes in humans [39]–[43].

Our data here indicate again that exome sequencing can rapidly identify genes causing dominant Mendelian diseases, which can occur in a heterozygous form. We further were able to identify this gene by sequencing exomes of only two affected patients and using available public databases, such as dbSNP131 and the 1000 Genomes Project. Additionally, the use of second-generation sequencing produces a high level of coverage, with subsequent higher accuracy, and allows more regions of a genome to be sequenced in a very cost effective manner. The 30-fold average coverage we obtained here is a very high sequencing depth. It covered 97% of the target sequence with ∼96% accuracy rate, and thus allowed us to identify variants with high confidence.

Using this technique, we successfully identified a gene for high myopia in an affected family. Several lines of evidence provided support for the mutations in *ZNF644*, and thus the mutated *ZNF644* gene, being the cause of high myopia: 1) only the S672G mutation identified in the two affected patients showed complete co-segregation with the disease phenotype in the family studied; 2) our analysis of the *ZNF644* gene in 300 unrelated, sporadic high myopia patients identified an additional three missense mutations and two mutations in the 3′ UTR which may affect mRNA stability or microRNA interaction; 3) none of these identified mutations were present in the 30 genomes of Han Chinese Beijing in the 1000 Genomes Project database, the Han Chinese Beijing SNPs in the dbSNP131 database, or 600 normal ethnicity-matched controls; and 4) Comparative analyses of *ZNF644* in other species showed that I587 is conserved and S672, R680, C699 are highly conserved among primates, placental animals, and other vertebrate species (http://genome.ucsc.edu/cgi-bin/hgPal). Based on protein structure, *ZNF644* is predicted to be a transcription factor (http://www.genecards.org/cgi-bin/carddisp.pl?gene=ZNF644), and given that it has potentially deleterious mutations in patients with high myopia, it may play a role in gene expression regulation in the retina and retinal pigment epithelium (RPE). One important issue in the genetic study of high myopia is the age of disease onset. We would have an informative censoring problem if family members of 951 did not show the disease phenotype because their age was too young. However, the disease phenotype studied in family 951 is very special. The disease onset was at 3–4 years old for all affected patients in the family 951 with high myopia; all affected patients developed high myopia by the age of seven. The youngest unaffected member in the family (V:2) is 9 years old now; he does not show any signs of myopia at all. In addition, all affected patients in the family had severe high myopia, which allowed us identify the affected patients easily. Therefore, there is very little chance that an unaffected family member does not show the trait by virtue of being too young.

Although it clearly has a ubiquitous level of expression, this is common for other genes involved in eye diseases (for example, the retinitis pigmentosa disease-causing gene *PRPC8* is ubiquitously expressed in human tissues [44]), and its expression in eye tissue allows for the *ZNF644* gene having activity in the eye. Note that, given that less than 4% of the sporadic high myopia cases had mutations in *ZNF644* (we identified 5 different mutations in 11 patients out of 300 cases), the *ZNF644* gene is unlikely to play a major role in sporadic high myopia.

*ZNF644* belongs to the Krüppel C2H2-type zinc-finger protein family, which contains 7 C2H2-type zinc fingers. Among the six identified mutations, four missense mutations were found clustered in exon 3 of the *ZNF644* gene, suggesting that this exon may code for important protein domain structures or have regulatory functions. The other two mutations were located in the 3′ UTR of *ZNF644* gene, which is a region often important for RNA degradation. The main feature of high myopia is axial elongation of the eye globe. Given that *ZNF644* is predicted to be a transcription factor that may regulate genes involved in eye development, a mutant ZNF644 protein may impact the normal eye development and therefore underlie the axial elongation of the eye globe in high myopia patients. However, the exact mechanism of ZNF644 action and its role in high myopia pathogenesis remains unclear, and future functional studies will be important. To date, there have been no documented studies on the *ZNF644* gene, and the data here indicating its involvement in a devastating eye disease provide excellent motivation for future investigation of the *ZNF644* gene, which in turn should enable dissection of its relationship with high myopia pathogenesis.

## Materials and Methods

### Ethics statement

All procedures used in this study conformed to the tenets of the Declaration of Helsinki. The Institutional Review Board and the Ethics Committee of Sichuan Academy of Medical Sciences & Sichuan Provincial People's Hospital approved the protocols used. Informed consent was obtained from all participants.

### Study population

We undertook exome sequencing and validation studies between September 1, 2009 and December 2, 2009. This study included a Han Chinese family (designated as 951) with high myopia that had 30 (20 living) family members (Figure 1, Table 1); 300 sporadic patients with high myopia; and 600 matched, normal controls. The 300 sporadic patients and the 600 controls were non-related, were of Han Chinese ethnicity (Figure 4, Table 4), came from the Chengdu region of Sichuan Province, China, and were recruited at the ophthalmic clinic at Sichuan Academy of Medical Sciences & Sichuan Provincial People's Hospital, Chengdu, China. All participants underwent an extensive, standardized examination by ophthalmologists, including visual acuity (VA) testing, a detailed clinical examination, optical coherence tomography (OCT), and ocular imaging prior to genetic testing. Refractive error and the radius of corneal curvature in the horizontal and vertical meridian were measured using an autorefractor (KR8800, Topcon, Tokyo, Japan). Final refractive error status was established with subjective visual acuity testing by trained and certified optometrists. The diagnosis for high myopia in this study required a spherical equivalent of ≤−6.0 DS for both eyes and an axial length of the eye globe of ≥26.0 mm for both eyes. For controls, the spherical equivalent for both eyes had to be in the range of −0.50 to +1.0 DS and show no evidence of disease in either eye.

### Targeted capture and exome sequencing

The exome sequencing approach was used to identify the disease-causing genetic variant for the high myopia family in the study (951). Genomic DNA was extracted from peripheral white blood cells, using Gentra Systems PUREGENE DNA purification kit (Minneapolis, MN, USA). Fifteen µg of genomic DNA from each of the two selected individuals (V:1 and III:2, Figure 1A) with high myopia from family 951 were separately sheared into about 200-bp DNA fragments by sonication. Exome capture was performed to collect the protein coding regions of human genome DNA using a NimbleGen 2.1M HD array as described in the manufacturer's instructions (Roche NimbleGen, Inc., Madison, WI, USA). The array was able to capture 18,654 (92%) of the 20,091 genes. The gene sequences for this array are available in the Consensus Coding Sequence Region (CCDS) database (http://www.ncbi.nlm.nih.gov/projects/CCDS/). The exon-enriched DNA libraries were then subjected to a second library construction in preparation for Illumina GA sequencing and were sequenced using the Illumina Genome Analyzer II platform, following the manufacturer's instructions (Illumina, San Diego, USA) [23]. We obtained a mean exome coverage of 30×, which allows each selected region of the genome to be checked, on average, 30 times. Such deep coverage provided sufficient depth to accurately call variants at ∼97% of each targeted exome.

### Read mapping and variant analysis

The human reference genome, together with its gene annotation, was downloaded from the UCSC database (http://genome.ucsc.edu/), version hg18 (build36). Alignment of the sequences from the two affected individuals was performed using SOAPaligner after we removed the duplicated reads [32], and SNPs were called using SOAPsnp set with the default parameters [33]. Indels affecting coding sequence or splicing sites were identified as described previously [34]. The thresholds for calling SNPs and short insertions or deletions (indels) included the following: 1) the number of unique mapped reads supporting a SNP had to be ≥4 and ≤100; and 2) the consensus quality score had to be ≥20 (The quality score is a Phred score, generated by the program SOAPsnp 12, quality score 20 represents 99% accuracy of a base call). The shared changes of the two affected individuals were obtained by further comparison of the variants of each of the two affected individuals. All changes were filtered against exome data of 30 genomes from ethnic Han Chinese individuals from Beijing available in the 1000 Genomes Project (February 28, 2011 releases for SNPs and February 16, 2011 releases for indel fttp://www.1000genome.org), and against the Han Chinese Beijing SNPs in the dbSNP131. Sanger sequencing was then used to validate the identified potential disease-causing variants. SIFT (http://sift.jcvi.org) was used to predict whether an amino acid substitution affects protein function.

### Mutation validation

All shared variants of the two affected individuals after filtering against the 30 Han Chinese Beijing genomes of 1000 Genomes Project and the Han Chinese Beijing SNPs in the dbSNP131 were then confirmed by direct polymerase chain reaction (PCR)-product sequencing using Bigdye terminator v3.1 cycle sequencing kits (ABI, Foster City, CA, USA) and analyzed on an ABI 3130XL Genetic Analyzer. We used Sanger sequencing to determine whether any of the remaining variants co-segregated with the disease phenotype in family 951 and used MLINK of the LINKAGE program to calculate a two-point LOD score for the detected variants to assess the locus position of the predicted disease gene in family 951 [35]. The primers flanking all exons of *ZNF644* were designed using primer 3 (http://frodo.wi.mit.edu/primer3/) (Table S1), and all exons of the 300 sporadic patients with high myopia were analyzed using the same method as above.

### ZNF644 expression

We looked at expression of the *ZNF644* gene in the human liver, retina, RPE, and placenta. The human liver, retina, and RPE were donated by a deceased 55-year-old Han Chinese male and the human placenta was donated by a 29-year-old Han Chinese female. Total RNA from the human liver, placenta, retina, and RPE was extracted by trizol (Invitrogen, Carlsbad, CA, USA), and reverse transcription was performed using a reverse transcription kit (Invitrogen, Carlsbad, CA, USA). The housekeeping gene glyceraldehyde-3-phosphate dehydrogenase (*GAPDH*) was used as an internal control (Table S2). All RT-PCR products were confirmed by direct sequencing.

## Supporting Information

Table S1 Primers for Sequencing Analysis of *ZNF644* Exons.
(DOC)

Table S2 Primers for gene expression by RT-PCR.
(DOC)
