# Supplementary material for: Exome Sequencing Identifies ZNF644 Mutations in High Myopia
Source: PLoS Genet. 2011 Jun 9;7(6):e1002084. doi: 10.1371/journal.pgen.1002084 (PMC3111487; doi:10.1371/journal.pgen.1002084)
Supplement: Table S1 — Primers for Sequencing Analysis of ZNF644 Exons. (DOC) [file pgen.1002084.s001.doc]

| **Table S1. Primers for Sequencing Analysis of *ZNF644* Exons** | | |
| --- | --- | --- |
| **Exon** | **Forward primer** | **Reverse primer** |
| Exon 1 | CGCTGCCCTCGTTTGTCT | CTTCCGCCACCCTCAGTC |
| Exon 2 | GGGGCACTTAGCAATAGT | ACTCCCATCAAATCAACTG |
| Exon 3-1 | TGTTTGTGTTCTTTCATTCT | GCTGTCCAGTGGTTAATG |
| Exon 3-2 | ATGCTGGTGCTCCTACTG | TCCCGTTACTGTATTGACA |
| Exon 3-3 | CTGCTTCAGTTGGTTGTGAC | AGGCATCCACAGATTCTAAGT |
| Exon 3-4 | GTGAAGCCTGAATCAACTG | ATTTCCTCCATCAACTTCTGT |
| Exon 3-5 | ACCAGGAGAGAAGACAGAAG | CTACCAAATGAATCAACACAT |
| Exon 3-6 | CCCTATGGTCACTTCTGATA | TTTTGAAATGCACAGGATAT |
| Exon 3-7 | CAAGCAGCAAAAGAAAAGTC | ATCTCATGCAAAAAGTTGTTA |
| Exon 3-8 | AAGAGCAAAGTGGAAGGTC | CACCACAGAGCTGACAAGT |
| Exon 4 | TTTTAGGGTGGTCAGTTTAT | TAACCAATCTGTGCTCTGT |
| Exon 5 | GGGAATAGGGAAATGAATG | CAGGCTGCTCTTGAACTC |
| Exon 6-1 | TGAATTGGGAGTTTTGATGT | CCCATTTTCCTGCTTTAGTA |
| Exon 6-2 | TGCCTCCATTACAGAAACTTC | TGCCCTATTTGAGTGAACAG |
| Exon 6-3 | CCCCACAAGACTTGCATAGA | CCTGTCCTGTAAGCATGTCA |
| Exon 6-4 | ACTCCAGTTGCATTTCTCAGA | CCAAGACACCTGCACCATAT |
