# Supplementary material for: Exome Sequencing Identifies ZNF644 Mutations in High Myopia
Source: PLoS Genet. 2011 Jun 9;7(6):e1002084. doi: 10.1371/journal.pgen.1002084 (PMC3111487; doi:10.1371/journal.pgen.1002084)
Supplement: Table S2 — Primers for gene expression by RT-PCR. (DOC) [file pgen.1002084.s002.doc]

| **Table S2. Primers for gene expression by RT-PCR** | | | |
| --- | --- | --- | --- |
| **Gene name** | **Gene id** | **Primer sequences** | **Product length** |
| ZNF644 | NM_201269 | Forward: CGTTCAGAAATGCGTTCTTCC | 255 |
|  |  | Reverse: TGTCGTTGTAAGTGCTTAATCC |  |
| GAPDH | NM_002046 | Forward: GAAGGTGAAGGTCGGAGTC | 226 |
|  |  | Reverse: GAAGATGGTGATGGGATTTC |  |
